# Frailty-related vulnerability and immune cell profiles in younger adults with schizophrenia: an exploratory study

**DOI:** 10.1186/s12888-026-08032-x

**Published:** 2026-04-11

**Authors:** Shan Hung, Shang-Ying Tsai, Chiou-Feng Lin, Kuo-Hsuan Chung

**Affiliations:** 1 Department of Psychiatry, Taipei Medical University Hospital, No.252, Wuxing St., Taipei, 11031 Taiwan; 2 Psychiatric Research Center, Taipei Medical University Hospital, Taipei, Taiwan; 3 Department of Psychiatry, School of Medicine, College of Medicine, Taipei Medical University, Taipei, Taiwan; 4 Graduate Institute of Medical Sciences, College of Medicine, Taipei Medical University, Taipei, Taiwan; 5 Department of Microbiology and Immunology, School of Medicine, College of Medicine, Taipei Medical University, Taipei, Taiwan; 6 Core Laboratory of Immune Monitoring, Office of Research & Development, Taipei Medical University, Taipei, Taiwan

**Keywords:** Frailty index, Schizophrenia, Immune aging, γ/δ T cells, Immune markers, Vulnerability, Exploratory

## Abstract

**Background:**

This exploratory study aimed to compare frailty characteristics between patients with schizophrenia and healthy controls, and to investigate the association between frailty severity and immune-inflammatory markers in individuals with schizophrenia.

**Methods:**

We recruited 27 community-dwelling patients with schizophrenia (mean age 39.9 ± 7.8 years) and 14 age-matched healthy controls (mean age 37.1 ± 8.4 years). Frailty was assessed using the Frailty Index-Laboratory (FI-Lab), the Edmonton Frail Scale (EFS), and the Clinical Frailty Scale (CFS). Immune-inflammatory markers were analyzed using flow cytometry. Statistical analyses examined group differences and associations with frailty severity.

**Results:**

Compared to healthy controls, patients with schizophrenia showed significantly higher scores on FI-Lab, EFS, and CFS. Several immune cell subsets, including memory (CD45RO^+^) γ/δ T^−^ Th17 and regulatory γ/δ T^−^ Th cells, were significantly elevated in the schizophrenia group. Within this group, FI-Lab scores were positively associated with memory (CD45RO^+^) γ/δ T^−^ Tc levels. No significant differences were observed for hs-CRP or homocysteine.

**Conclusions:**

Our findings suggest that younger patients with schizophrenia may exhibit early signs of frailty and altered immune cell profiles. While limited by sample size and cross-sectional design, these findings highlight potential immune correlates of frailty-related vulnerability in this population.

**Clinical trial registration:**

Not applicable.

**Supplementary information:**

The online version contains supplementary material available at 10.1186/s12888-026-08032-x.

## Introduction

Schizophrenia affects approximately 24 million people worldwide, corresponding to about 1 in 300 individuals, and is characterized by early onset, a chronic course, frequent relapse, and progressive functional deterioration, resulting in substantial health, social, and economic burdens [1]. Accumulating evidence suggests that individuals with schizophrenia experience accelerated physical aging compared with the general population. Large-scale epidemiological studies have reported a standardized all-cause mortality ratio of approximately 2.6 [2], with an estimated reduction in life expectancy of 20–23 years compared with the general population [3]. Proposed mechanisms underlying this excess morbidity and mortality include cognitive decline, metabolic dysregulation, neurodevelopmental alterations extending beyond the perinatal period, oxidative stress–related cellular damage [4], long-term pharmacological treatment, chronic psychosocial stress, and adverse health behaviors [5]. Despite growing interest in accelerated aging in schizophrenia, empirical data on biological markers of aging remain limited, and the extent to which schizophrenia is associated with early vulnerability or frailty across adulthood has not been fully elucidated [5, 6].

Frailty is conceptually defined as a clinically recognizable state of increased vulnerability resulting from a decline in physiological reserve and function across multiple organ systems, leading to reduced resilience to everyday or acute stressors [7]. Although frailty has traditionally been studied in older populations, it is increasingly recognized as a multidimensional construct rather than a condition confined to late life. Frailty is also associated with substantial healthcare costs; for example, older adults with frailty incur approximately fivefold higher short-term healthcare expenditures compared with non-frail counterparts [8]. Several conceptual and operational approaches have been developed to assess frailty [9, 10]. The frailty phenotype model focuses on physical characteristics such as weakness and slowness [11, 12], whereas the frailty index (FI) model, based on deficit accumulation, quantifies vulnerability by integrating a broad range of health deficits, including symptoms, clinical conditions, and laboratory abnormalities [13, 14] Mixed models, such as the Tilburg Frailty Indicator and the Edmonton Frail Scale, further incorporate psychosocial and functional domains into frailty assessment [15, 16]. Compared with phenotype-based approaches, the FI framework provides a graded and continuous measure of vulnerability and has demonstrated sensitivity in predicting adverse outcomes, including hospitalization and mortality, across a wide age range [17–19]. Laboratory-based frailty indices (FI-Lab), derived from routinely available clinical tests, have been proposed as indicators of subclinical physiological dysregulation and early vulnerability rather than categorical frailty syndromes [20, 21]. Importantly, psychosocial and functional factors may modulate frailty trajectories, particularly in populations with severe mental illness, where social isolation, unemployment, and cognitive impairment are prevalent. These factors may either exacerbate or partially buffer the progression of vulnerability and therefore warrant consideration when frailty is examined in schizophrenia.

Frailty is increasingly conceptualized as a cross-lifespan condition that can emerge in early or mid-adulthood, particularly among individuals exposed to chronic disease or long-term stressors [22]. Aging-related paradigms such as “inflamm-aging,” “oxi-inflamm-aging,” and “inflamm-inactivity,” primarily described in aging populations, emphasize chronic low-grade inflammation as a shared biological pathway contributing to vulnerability and functional decline [7]. Genetic and epigenetic susceptibility, metabolic disturbances, lifestyle factors, and chronic medical or psychiatric illness may all contribute to these processes. However, empirical studies examining biological correlates of frailty-related vulnerability in younger adults, particularly those with schizophrenia, remain scarce. Clarifying whether immune-inflammatory alterations are associated with early frailty-related features may inform future risk stratification and preventive strategies across adulthood.

Accordingly, the present exploratory cross-sectional study aimed to examine frailty-related characteristics and immune-inflammatory profiles in community-dwelling adults with schizophrenia aged 20–55 years, compared with age-matched healthy controls. We employed three complementary frailty measures—the Frailty Index–Laboratory (FI-Lab), the Edmonton Frail Scale, and the Clinical Frailty Scale—to capture biological, functional, and psychosocial dimensions of vulnerability. In addition, we explored associations between frailty severity and peripheral immune cell subsets assessed by flow cytometry. Given the exploratory nature and cross-sectional design of this study, causal inferences cannot be drawn; however, our findings may generate hypotheses regarding early biological vulnerability and immune alterations associated with frailty-related risk in schizophrenia.

## Methods

### Patient and control selection

This exploratory cross-sectional study enrolled 27 adults with schizophrenia who were recruited from outpatient psychiatric clinics and day-care units of the three affiliated hospitals of Taipei Medical University Hospital. All patients met the Diagnostic and Statistical Manual of Mental Disorders, Fifth Edition (DSM-5) criteria for schizophrenia, as confirmed by board-certified psychiatrists through clinical interviews and review of medical records.

Eligible participants were required to be between 20 and 55 years of age in order to focus on frailty-related vulnerability in early to mid-adulthood while minimizing confounding effects related to advanced aging. Exclusion criteria included intellectual disability, significant cognitive impairment or illiteracy that could interfere with assessment procedures, major medical illnesses accompanied by neurocognitive disorders, current alcohol use disorder or illicit substance use disorder, substance-induced psychiatric disorders, or psychiatric disorders secondary to another medical condition. Individuals with active suicidal or homicidal ideation at the time of assessment were also excluded. Female participants who were pregnant or lactating were excluded to avoid potential physiological confounding.

To minimize the potential influence of recent lifestyle changes on biological and frailty-related measures, participants were excluded if they reported substantial changes in dietary habits or physical activity patterns within the four weeks preceding enrollment. In addition, all patients were required to have a stable antipsychotic treatment regimen for at least four weeks prior to participation to reduce the confounding effects of recent medication adjustments on immune-inflammatory markers and laboratory parameters.

### Study procedure

All participants were enrolled and evaluated at a single time point as part of this exploratory cross-sectional study. Upon enrollment, basic demographic and physical health data were collected, including age, sex, height, weight, body mass index (BMI), waist circumference, and vital signs (blood pressure, heart rate, and respiratory rate). Information on smoking status was also collected as part of lifestyle-related variables. Additional sociodemographic variables, including years of education and current or previous employment status, were also recorded.

In addition to demographic information, laboratory data required for the Frailty Index–Laboratory (FI-Lab) and immune-inflammatory profiling were obtained. Participants also completed a series of standardized clinical assessments to evaluate frailty status and severity, as well as biopsychosocial characteristics relevant to schizophrenia. These assessments encompassed measures of physical, functional, and psychosocial domains.

Data were obtained directly from participants through structured interviews and standardized rating scales, supplemented by review of available medical and psychiatric records to enhance data accuracy and completeness. To reduce participant burden and minimize mental fatigue, all assessments and measurements were designed to be completed within approximately 45–60 minutes. Multiple sources of information, including self-report, clinical interviews, and chart review, were used to verify key clinical and demographic variables.

### Data collection tools

Clinical assessments in the present study encompassed frailty-related vulnerability, psychiatric symptom severity, health-related quality of life, subjective well-being, and medical comorbidity. Frailty was evaluated using complementary instruments reflecting biological, physical, and psychosocial domains, consistent with the frailty index framework and mixed physical–psychosocial models.

Frailty-related measures included the Edmonton Frail Scale (EFS), the Clinical Frailty Scale (CFS), and the Frailty Index–Laboratory (FI-Lab). The EFS is a multidimensional instrument incorporating physical, cognitive, and psychosocial components [16]. The CFS is a clinician-rated scale emphasizing functional status and physical frailty [17, 23]. The FI-Lab is a deficit accumulation–based index derived from routine laboratory variables and physiological parameters, reflecting subclinical biological vulnerability rather than categorical frailty status [22]. An FI-Lab score was calculated only when more than 70% of the predefined laboratory variables were available for a given participant.

For descriptive purposes, operational thresholds commonly used in the literature were applied to characterize frailty syndrome, defined as an EFS score ≥ 8, a CFS score ≥ 5, and an FI-Lab score ≥ 0.25 [16, 17, 22, 23]. Given the relatively young age of the study population, these thresholds were not intended to indicate geriatric frailty per se but were used to facilitate comparison with prior studies and to contextualize frailty-related vulnerability across instruments.

Psychiatric symptom severity in participants with schizophrenia was assessed using the Positive and Negative Syndrome Scale (PANSS), which has been validated in a Mandarin Chinese version [24]. Overall illness severity at the time of assessment was rated by clinicians using the Clinical Global Impression (CGI) scale [25]. Health-related quality of life was evaluated using the 12-Item Short Form Health Survey (SF-12), including physical and mental component summaries [26]. Subjective well-being was assessed using the 5-item World Health Organization Well-Being Index (WHO-5) [27]. Medical comorbidity burden was quantified using the Charlson Comorbidity Index [28].

All assessment instruments used in this study have been previously validated and demonstrated acceptable reliability in Taiwanese populations.

### Blood sample collection and analysis

#### Laboratory measures for FI-Lab

To construct the Frailty Index–Laboratory (FI-Lab), routine blood tests were obtained using standard clinical laboratory procedures. The FI-Lab was derived from 21 laboratory variables, excluding systolic and diastolic blood pressure, in accordance with established methods [20, 21]. These variables included serum albumin, aspartate aminotransferase (AST), calcium, creatinine, folate, red blood cell count, fasting glucose, hemoglobin, mean corpuscular volume, alkaline phosphatase, inorganic phosphorus, potassium, total protein, sodium, thyroid-stimulating hormone, total thyroxine (T4), free thyroxine (free T4), urea, Venereal Disease Research Laboratory test (VDRL), vitamin B12, and white blood cell count. An FI-Lab score was calculated only when more than 70% of the predefined laboratory variables were available for a given participant.

In addition, high-sensitivity C-reactive protein (hs-CRP) and homocysteine levels were measured as general indicators of systemic inflammation, based on prior literature linking these biomarkers to frailty and cardiometabolic risk.

Each laboratory variable was dichotomized as 0 (within the reference range) or 1 (outside the reference range) based on standardized clinical laboratory reference intervals routinely used in Taipei Medical University Hospital clinical laboratories. The FI-Lab score was calculated as the proportion of abnormal variables divided by the total number of available variables for each participant. Only participants with at least 70% of laboratory data available were included in FI-Lab calculations.

#### Immunophenotyping and immune cell profiling

Peripheral immune cell profiles were assessed using flow cytometry–based immunophenotyping performed by the Immune Monitoring Core of Taipei Medical University. This exploratory immune profiling approach was designed to characterize overall patterns of immune cell distribution, differentiation, and activation, rather than to test predefined mechanistic hypotheses.

Approximately 5 mL of peripheral venous blood was collected from each participant into heparinized tubes (BD Biosciences, San Jose, CA, USA). Peripheral blood mononuclear cells (PBMCs) were isolated from buffy coats using SepMate™ tubes (STEMCELL Technologies, Vancouver, Canada) according to the manufacturer’s instructions. A combination of no-wash, no-lyse procedures and conventional staining protocols was applied to optimize cell recovery and minimize processing-related variability. Following washing with phosphate-buffered saline, Fc receptors were blocked using a human Fc receptor–blocking reagent (Miltenyi Biotec, Germany) for 30 minutes at 4 °C, after which cells were surface-stained with fluorochrome-conjugated monoclonal antibodies.

Antibody panels included markers for major immune cell populations, T-cell lineages, and differentiation or activation status, including CD3, CD4, CD8, CD11c, CD14, CD16, CD19, CD25, CD28, CD45RA, CD45RO, CD56, CD62L, TCRγδ, HLA-DR, CCR3, CCR5, CCR6, CCR10, and CXCR3 (Thermo Fisher Scientific, MA, USA). Flow cytometric acquisition was performed using a three-laser Attune™ NxT flow cytometer (Life Technologies, Carlsbad, CA, USA), and data were analyzed using manufacturer-provided software.

#### Gating strategy and immune subsets

Gating strategies were applied in a standardized, stepwise manner. Initial gates were defined using forward scatter (FSC) and side scatter (SSC) properties to exclude debris and non-cellular events and to define circulating mononuclear cells. Major immune cell populations—including T cells, B cells, natural killer (NK) cells, NKT cells, monocytes, and dendritic cells—were subsequently identified based on established surface marker combinations, as detailed in the Supplemental Data.

Within the T-cell compartment, γδ T cells were identified as a distinct lineage based on TCRγδ expression. Analyses of functional T-helper (Th) and cytotoxic T-cell (Tc) subsets were restricted to γδT− (αβ) T cells. CD4^+^ and CD8^+^ αβ T cells were further characterized according to chemokine receptor expression patterns, including CXCR3, CCR3, CCR5, CCR6, and CCR10, corresponding to Th1/Tc1, Th2/Tc2, Th17/Tc17, Th22/Tc22, and regulatory Th/Tc–like phenotypes.

T-cell differentiation and activation status were assessed using CD45RA, CD45RO, CD62L, CD28, and HLA-DR expression within CD4^+^, CD8^+^, and γδ T-cell populations. Definitions of all immune cell subsets and corresponding gating regions are summarized in the Supplemental Data.

#### Frailty-related immune markers

Based on prior literature linking immunosenescence and frailty to alterations in T-cell differentiation and activation profiles, a subset of immune parameters was designated a priori as frailty-related immune markers. These parameters were selected to capture immune activation states and differentiation patterns that may reflect broader biological vulnerability rather than disease-specific immune mechanisms.

Frailty-related immune markers included the proportions of CD4^+^ and CD8^+^ T cells expressing CD28, overall CD4^+^ and CD8^+^ T-cell distributions, total CCR5^+^ T cells, and CCR5^+^ CD8^+^ T-cell subsets. In addition, CCR5^+^ CD45RO^−^ (naïve) T-lymphocyte subsets were examined to reflect early differentiation status within the T-cell compartment. Immune cell subset values were expressed as percentages of total mononuclear cells. Given the exploratory nature of the study, analyses focused on relative distributions of immune cell populations rather than absolute cell counts.

### Statistical methods

All statistical analyses were performed using the Statistical Package for the Social Sciences (SPSS) for Windows, version 17.0 (SPSS Inc., Chicago, IL, USA). Descriptive statistics were summarized as means and standard deviations for continuous variables and as frequencies and percentages for categorical variables.

Group comparisons between participants with schizophrenia and healthy controls were conducted using independent-samples t tests for normally distributed variables and Mann–Whitney U tests for non-normally distributed variables. Categorical variables were compared using Pearson’s chi-square test or Fisher’s exact test, as appropriate. Normality was assessed using the Kolmogorov–Smirnov test and visual inspection.

Associations between frailty severity, as measured by the Frailty Index–Laboratory (FI-Lab), and clinical or immune-inflammatory variables were examined using linear regression analyses. Covariates considered included age, sex, waist circumference (as a proxy for metabolic status), smoking status, age at illness onset, and antipsychotic burden (chlorpromazine-equivalent dose). Given the limited sample size, only a subset of covariates was included in the final model to avoid overfitting.

A total of approximately 30 immune cell subsets were examined in exploratory analyses. Given the exploratory nature of the study and the limited sample size, no formal correction for multiple comparisons (e.g., false discovery rate) was applied. Therefore, the results should be interpreted as hypothesis-generating rather than confirmatory. All statistical tests were two-tailed, and a p value of <0.05 was considered statistically significant.

## Results

### Descriptive variables

Table 1 summarizes the sociodemographic and clinical characteristics of participants with schizophrenia and healthy controls. The study sample comprised 19 men (46.3%) and 22 women (53.7%), with a mean age of 38.9 ± 8.0 years. No significant between-group differences were observed in age (*p* = 0.58), sex distribution (*p* = 0.606), or body mass index (BMI; *p* = 0.59). These findings indicate that the groups were comparable in key demographic variables, including age, sex, and BMI.

**Table 1:** Sociodemographic and physical health characteristics of participants with schizophrenia and healthy controls

	Patients with schizophrenia (*n* = 27)	Healthy controls (*n* = 14)	*p* value
Age (years)	39.9 ± 7.8	37.1 ± 8.4	0.293
Sex (%)	Male: 14 (51.9%) Female: 13 (48.1%)	Male: 5 (35.7%) Female: 9 (64.3%)	0.338
BMI (kg/m^2^)	24.8 ± 5.1	24.0 ± 3.4	0.588
**Waist circumference (cm)**	87.8 ± 13.0	72.0 ± 14.0	0.001
**Marital status (%)**	Married: 1(4.5%) Others: 21 (95.5%)	Married: 5 (35.7%) Others: 9 (64.3%)	0.041
Living arrangement (%)	Living with family: 15 (83.3%) Others: 3 (16.7%)	Living with family: 11 (84.6%) Other: 2 (15.4%)	0.166
**Years of education**	13.8 ± 2.2	15.9 ± 2.2	0.008
**Employment status**	Employed: 5 (33.3%) Unemployed: 10 (66.7%)	Employed: 14 (100%) Unemployed: 0 (0%)	<0.001

Percentages for categorical variables represent proportions within each group. Continuous variables are expressed as means ± standard deviations. Group comparisons were conducted using independent t-tests or Mann–Whitney U tests for continuous variables, and Pearson’s chi-square or Fisher’s exact tests for categorical variables. Abbreviations: BMI, Body Mass Index; cm, centimeter

Compared with healthy controls, participants with schizophrenia had a significantly greater waist circumference (*p* = 0.001) and fewer years of education (*p* = 0.008). They were also less likely to be married (*p* = 0.014) and less likely to be employed (*p* < 0.001). No significant difference in living arrangement was observed between the two groups.

Among participants with schizophrenia, antipsychotic treatment patterns were as follows: 25.9% were receiving clozapine, 22.2% paliperidone, 11.1% risperidone, 11.1% quetiapine, 11.1% olanzapine, 7.4% aripiprazole, and 11.1% a combination of antipsychotic medications.

In exploratory analyses including all participants, linear regression models were used to examine associations between Frailty Index–Laboratory (FI-Lab) scores and selected demographic and clinical variables. Age (*p* = 0.323), waist circumference (*p* = 0.549), psychotropic medication use (*p* = 0.230), and smoking status (*p* = 0.995) were not significantly associated with FI-Lab scores.

### Comparison of rating scale results of schizophrenia and control groups

Table 2 presents comparisons of frailty indices and related rating scale scores between participants with schizophrenia and healthy controls. Participants with schizophrenia exhibited significantly higher frailty scores across all three frailty measures, including the Frailty Index–Laboratory (FI-Lab; *p* = 0.037), the Edmonton Frail Scale (EFS; *p* < 0.001), and the Clinical Frailty Scale (CFS; *p* < 0.001), compared with healthy controls.

**Table 2:** Comparison of frailty indices and rating scale scores between participants with schizophrenia and healthy controls

	Patients with schizophrenia	Healthy controls	*p* value
**FI-Lab**	0.079 ± 0.067	0.036 ± 0.044	**0.037**
**EFS**	3.33 ± 2.78	0.64 ± 0.50	**<0.001**
**CFS**	3.44 ± 0.97	2.00 ± 0.68	**<0.001**
**WHO 5**	60.44 ± 21.38	74.86 ± 14.33	**0.029**
**SF-12 Physical Component Summary (PCS)**	44.88 ± 7.11	52.58 ± 4.75	**0.001**
SF-12 Mental Component Summary (MCS)	47.11 ± 11.58	48.93 ± 6.16	0.515

Frailty was assessed using the Frailty Index–Laboratory (FI-Lab), the Edmonton Frail Scale (EFS), and the Clinical Frailty Scale (CFS). Clinical measure included the Clinical Global Impression (CGI). Group comparisons were conducted using independent t-tests or Mann–Whitney U tests based on data distribution. All variables are continuous. Significant differences (*p* < 0.05) are indicated in bold. Abbreviations: FI-Lab, Frailty Index–Laboratory; EFS, Edmonton Frail Scale; CFS, Clinical Frailty Scale; WHO-5, WHO (Five) Well-Being Index; SF-12, 12-Item Short Form Health Survey; PCS, physical component summary; MCS, mental component summary

Although none of the participants in either group met the conventional FI-Lab threshold for frailty (FI-Lab ≥ 0.25), mean FI-Lab scores were significantly higher in the schizophrenia group, indicating greater biological vulnerability on a continuous scale. In contrast, categorical frailty classifications based on functional and mixed-domain measures identified a subset of participants with schizophrenia as frail: 11.1% (*n* = 3) met the EFS criterion (EFS ≥ 8), and 18.5% (*n* = 5) met the CFS criterion (CFS ≥ 5), whereas no participants in the control group met frailty criteria on either scale.

With respect to health-related quality of life and well-being, participants with schizophrenia reported significantly lower scores on the World Health Organization Well-Being Index (WHO-5; *p* = 0.029) and on the physical component summary of the 12-Item Short Form Health Survey (SF-12 PCS; *p* = 0.001). No significant between-group difference was observed for the mental component summary of the SF-12 (SF-12 MCS; *p* = 0.515).

### Comparison of immune-inflammatory markers of schizophrenia and control groups

**Fig. 1:**
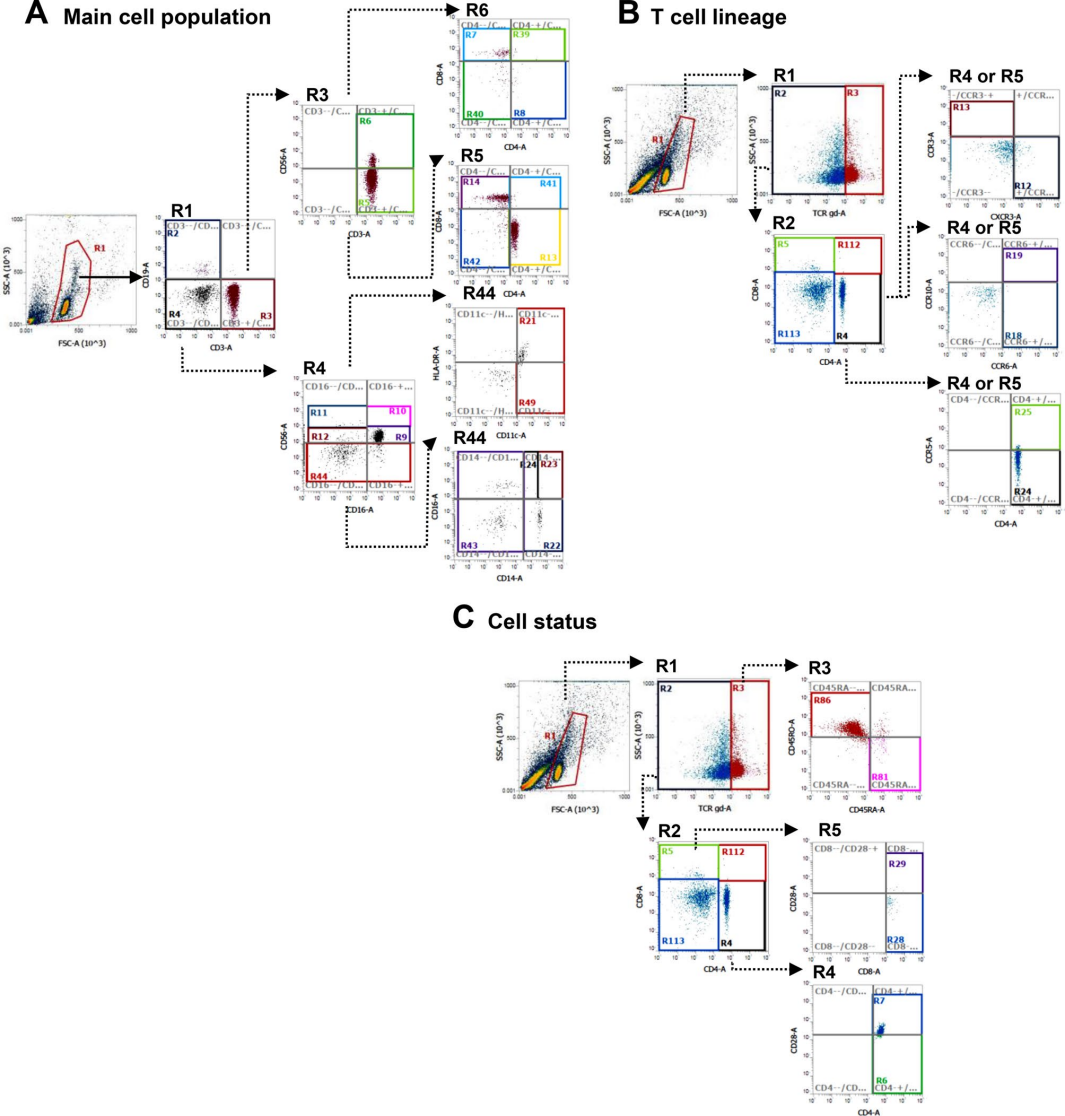
Flow cytometric gating strategy used for the analysis of peripheral blood immune cell subsets. Representative data from one participant are shown for main immune cell populations (**A**), T cell lineage subsets (**B**), and T cell differentiation status (**C**). Circulating mononuclear cells (MNCs) were initially gated on a forward scatter (FSC) versus side scatter (SSC) plot. Major immune cell populations, including lymphocytes (B and T cells), natural killer (NK) cells, NKT cells, monocytes, and dendritic cells, were identified based on the expression of CD3, CD4, CD8, CD11c, CD14, CD16, CD19, CD56, and HLA-DR. T cell lineage subsets were characterized within γδT− (αβ) CD4+ and CD8+ T cells using chemokine receptor expression (CXCR3, CCR3, CCR5, CCR6, and CCR10). γδ T cells were analyzed as a separate lineage. T cell differentiation and activation status was assessed using CD45RA, CD45RO, and CD28 expression within CD4+, CD8+, and γδ T cell populations. The expression profiles of all immune cell subsets were analyzed according to predefined marker combinations and are summarized in the Supplemental Data

Figure 1 illustrates the flow cytometric gating strategy used to identify major immune cell populations and T-cell subsets. Quantitative results for immune-inflammatory markers are summarized in Table 3.

**Table 3:** Comparison of immune-inflammatory markers between participants with schizophrenia and healthy controls

	Patients with schizophrenia	Healthy controls	*p* value
hs-CRP	0.30 ± 0.45	0.13 ± 0.17	0.200
Homocysteine	11.08 ± 4.56	8.95 ± 2.76	0.090
**CD56^dim**	1.962 ± 1.6504	3.661 ± 3.7533	**0.045**
**Memory (CD45RO+) γ/δ T− Th17**	0.798 ± 0.9320	0.226 ± 0.2794	**0.010**
**Naïve (CD45RA+) γ/δ T+**	1.436 ± 1.6648	0.654 ± 0.4726	**0.043**
**Regulatory γ/δ T− Th**	1.461 ± 1.3672	0.633 ± 0.7526	**0.023**
**Memory (CD45RO+) Regulatory γ/δ T− Th**	0.414 ± 0.4645	0.104 ± 0.0849	**0.005**
**Naïve (CD45RA**^**+**^**) γ/δ T**^**−**^** Tc**	2.479 ± 2.5317	1.276 ± 0.8773	**0.046**

Immune-inflammatorymarkers were compared between participants with schizophrenia and healthycontrols using independent t-tests or Mann–Whitney U tests, depending on thedistribution of each variable. Significant differences (*p* < 0.05) are indicatedin bold. All variables are continuous and were derived from flow cytometricanalyses

No significant between-group differences were observed in circulating levels of high-sensitivity C-reactive protein (hs-CRP; *p* = 0.200) or homocysteine (*p* = 0.090). In addition, no significant differences were identified in the proportions of B cells, monocytes, or dendritic cells between participants with schizophrenia and healthy controls.

Exploratory analyses of lymphocyte subsets revealed several differences between groups. Participants with schizophrenia exhibited significantly higher proportions of memory (CD45RO^+^) γ/δ T^−^ Th17 cells (*p* = 0.010), naïve (CD45RA^+^) γ/δ T^+^ cells (*p* = 0.043), regulatory γ/δ T^−^ Th cells (*p* = 0.023), memory (CD45RO^+^) regulatory γ/δ T^−^ Th cells (*p* = 0.005), and Naïve (CD45RA^+^) γ/δ T^−^ cytotoxic T cells (Tc) (*p* = 0.046), compared with healthy controls. In contrast, the proportion of CD56^dim cells was significantly lower in the schizophrenia group (*p* = 0.045).

All immune cell subset values were expressed as percentages of total mononuclear cells. Given the exploratory nature of these analyses and the number of immune parameters examined, these findings should be interpreted as descriptive patterns of immune cell distribution rather than definitive evidence of specific immunological mechanisms.

### Correlation between FI-Lab results and immune-inflammatory markers

Table 4 summarizes the results of exploratory linear regression analyses examining associations between Frailty Index–Laboratory (FI-Lab) scores and selected biochemical, clinical, and immune-inflammatory variables in participants with schizophrenia. Among the examined immune cell subsets, memory (CD45RO^+^) γδT− cytotoxic T cells were significantly associated with FI-Lab scores and were therefore selected for exploratory regression analysis based on observed between-group differences and biological relevance.

**Table 4:** Multivariable linear regression analysis of factors associated with FI-Lab scores in participants with schizophrenia

Independent Variables	β	95% CI for β	*p* value	
Age	0.000	−0.003–0.003	0.865	
Waist circumference	−0.001	−0.003–0.001	0.192	
Age of disease onset	0.001	−0.003–0.005	0.650	
CPZ equivalent dose	−0.000017	not shown due to rounding	0.624	
**Memory (CD45RO+) γ/δ T− Tc**	0.026	0.012–0.039	**0.001**	

Multivariable linear regression analysis of factors associated with FI-Lab scores in participants with schizophrenia. β represents unstandardized regression coefficients. Significant predictors are highlighted in bold. CPZ: chlorpromazine equivalent dose

In contrast, demographic and clinical variables, including age, sex, waist circumference, age at illness onset, and antipsychotic burden expressed as chlorpromazine-equivalent dose, were not significantly associated with FI-Lab scores in this sample.

Given the exploratory nature of the analysis and the limited sample size, these findings are presented as preliminary associations rather than evidence of causal or predictive relationships.

## Discussion

The present exploratory cross-sectional study examined frailty-related vulnerability and immune-inflammatory profiles in community-dwelling adults with schizophrenia aged 20–55 years, compared with age-matched healthy controls. Using complementary frailty measures capturing biological, functional, and psychosocial dimensions, we observed higher frailty-related scores across multiple measures. In addition, exploratory immune profiling revealed alterations in specific T-cell subsets that were associated with frailty-related biological vulnerability as measured by the Frailty Index-Laboratory (FI-Lab).

Across all three frailty instruments, including the FI-Lab, Edmonton Frail Scale (EFS), and Clinical Frailty Scale (CFS), participants with schizophrenia demonstrated higher frailty scores than healthy controls. Notably, none of the participants in either group met the conventional FI-Lab threshold for frailty, defined as a score of 0.25 or higher. In contrast, a subset of participants with schizophrenia met frailty criteria based on the EFS and CFS. This pattern suggests that, in this relatively young cohort, schizophrenia is associated with subclinical biological vulnerability and functional frailty features rather than overt geriatric frailty. The FI-Lab, which reflects accumulated laboratory abnormalities, may be particularly sensitive to early physiological dysregulation and vulnerability across adulthood, even when categorical frailty thresholds are not reached [20, 21]. Functional and mixed-domain measures, on the other hand, may capture psychosocial and activity-related deficits that emerge earlier in the course of schizophrenia [16, 17]. Given the relatively young age range of this cohort (20–55 years), frailty measures in this study should not be interpreted as reflecting geriatric frailty. Instead, they likely represent early vulnerability and subclinical physiological dysregulation. This conceptualization is consistent with emerging evidence that frailty can be understood as a continuum across the lifespan, particularly in populations with chronic medical or psychiatric conditions.

The finding that FI-Lab scores differed between groups on a continuous scale but did not reach categorical frailty thresholds is consistent with prior population-based studies demonstrating that laboratory-based frailty indices are associated with graded health risks across the life course, including in non-elderly adults [21, 22]. These results support the conceptualization of frailty as a continuum of vulnerability rather than a dichotomous condition, particularly in populations exposed to chronic psychiatric illness and long-term psychosocial stressors [7].

With respect to immune-inflammatory markers, no significant between-group differences were observed in circulating high-sensitivity C-reactive protein or homocysteine levels. This contrasts with prior studies linking frailty to elevated inflammatory markers, particularly in older adults and medically ill populations [29, 30]. The absence of differences in these markers in the present study may reflect the relatively young age, clinical stability, and community-dwelling status of the participants, as well as limited statistical power. These findings suggest that frailty-related biological vulnerability in schizophrenia may be associated with subtle alterations in immune cell distribution; however, these observations should be interpreted cautiously given the exploratory design [31].

In contrast, exploratory immunophenotyping revealed differences in several T-cell subsets between participants with schizophrenia and healthy controls. Specifically, higher proportions of memory and regulatory αβ T-helper subsets, along with altered γδ T-cell and cytotoxic T-cell distributions were observed in the schizophrenia group, while CD56^dim cell proportions were lower. Furthermore, among participants with schizophrenia, the proportion of memory CD45RO-positive gamma-delta-negative cytotoxic T cells was associated with FI-Lab scores. These findings should be interpreted cautiously, as the immune analyses were exploratory and not designed to test specific mechanistic hypotheses. Rather than indicating a definitive immunological pathway, these results suggest that frailty-related biological vulnerability in schizophrenia may be accompanied by subtle shifts in immune cell differentiation and activation patterns, which are broadly consistent with concepts of immunosenescence and inflamm-aging described in the aging literature [32–34].

Importantly, no significant differences were observed in B cells, monocytes, or dendritic cells, underscoring the selective and non-global nature of the immune alterations detected. Given the number of immune parameters examined and the limited sample size, these findings should be viewed as hypothesis-generating signals that warrant replication in larger, longitudinal studies incorporating absolute cell counts and functional immune assays [35, 36].

Several limitations of the present study should be acknowledged. First, the cross-sectional design precludes conclusions regarding temporal or causal relationships between frailty-related vulnerability and immune alterations. Second, the modest sample size limits statistical power, particularly in analyses involving multiple immune markers, increasing the risk of both type I and type II errors. Third, given the number of immune parameters examined and the absence of correction for multiple comparisons, the findings should be considered exploratory and hypothesis-generating. Fourth, although antipsychotic burden was accounted for using chlorpromazine-equivalent doses and treatment stability was required, residual medication effects cannot be fully excluded [5]. In addition, smoking status and metabolic factors, including waist circumference as a proxy for central obesity, were considered; however, residual confounding related to lifestyle and metabolic status may still influence the observed associations. Finally, the study population consisted of relatively young, clinically stable, community-dwelling individuals, which may limit generalizability to older or more severely ill populations with schizophrenia.

Despite these limitations, this study has several strengths, including the use of multiple complementary frailty measures, the focus on a younger schizophrenia population, and the integration of immune cell profiling with laboratory-based frailty assessment. Together, these findings suggest that adults with schizophrenia may exhibit early frailty-related vulnerability and accompanying immune alterations prior to the development of overt frailty. Future longitudinal studies with larger samples are needed to clarify the temporal dynamics of frailty progression, to validate immune correlates of biological vulnerability, and to determine whether early identification of frailty-related risk may inform preventive or rehabilitative interventions in schizophrenia [6].

## Electronic supplementary material

Below is the link to the electronic supplementary material.

Supplementary Material 1

## Data Availability

The datasets generated and/or analysed during the current study are not publicly available due to privacy restrictions but are available from the corresponding author on reasonable request.
